# Serologic and behavioral risk survey of workers with wildlife contact in China

**DOI:** 10.1371/journal.pone.0194647

**Published:** 2018-04-03

**Authors:** Corina Monagin, Blanca Paccha, Ning Liang, Sally Trufan, Huiqiong Zhou, De Wu, Bradley S. Schneider, Aleksei Chmura, Jonathan Epstein, Peter Daszak, Changwen Ke, Peter M. Rabinowitz

**Affiliations:** 1 Metabiota Inc., San Francisco, California, United States of America; 2 Yale University, New Haven, Connecticut, United States of America; 3 University of Washington Center for One Health Research, Seattle, Washington, United States of America; 4 Guangdong Provincial Center for Disease Control and Prevention, Guangzhou, Guangdong, China; 5 EcoHealth Alliance, New York, United States of America; Hokkaido University Graduate School of Medicine, JAPAN

## Abstract

We report on a study conducted in Guangdong Province, China, to characterize behaviors and perceptions associated with transmission of pathogens with pandemic potential in highly exposed human populations at the animal-human interface. A risk factor/exposure survey was administered to individuals with high levels of exposure to wildlife. Serological testing was performed to evaluate prior infection with several wildlife viral pathogens. Follow up serology was performed on a subset of the cohort as well as close contacts of individuals. 1,312 individuals were enrolled in the study. Contact with a wide range of wildlife species was reported in both occupational and occasional contexts. The overall proportion of individuals seropositive to any of the tested wildlife pathogens was approximately 4.0%. However, persons employed as butchers demonstrated a seropositivity of 9.0% to at least one pathogen of interest. By contrast, individuals working as hunters had lower rates of seropositivity. Among the study population, a number of other behaviors showed correlation with seropositivity, including contact with particular wildlife species such as field rats. These results demonstrate the need to further explore zoonotic risks of particular activities regarding wildlife contact, and to better understand risks of persons working as butchers with wildlife species.

## Introduction

The majority of human infectious diseases have an animal origin, therefore understanding the human-animal interface as it relates to disease emergence and risk is of upmost importance [1]. The increasing frequency and variety of human-wildlife interactions in China provide opportunities for the transmission of zoonotic pathogens from animals to humans [2]. Handling, transporting, and butchering of hunted or farmed wildlife poses a risk of pathogen spillover into humans [3]. In southern China provinces, including Guangdong, a significant percentage of the population obtains fresh meat for consumption from wet markets, community markets that specialize in selling and butchering live animals, including animals that are rare and endangered. Research has demonstrated that human-animal interfaces, such as within these wet markets, provide an ideal environment for infectious disease emergence, transmission, and amplification [4–7].

Wet markets and restaurants that butcher and sell wild animals are common in China and South East Asia, creating a high-risk interface where humans come into regular contact with the blood and bodily fluids of wild and domesticated animals. Intermingling of wildlife in wet markets can lead to inter-species transmission of pathogens, amplifying and maintaining these pathogens, and may result in spillover into a species that can more efficiently transmit the pathogen to humans [8]. Furthermore, increasing numbers of individuals are traveling to urban-rural interface areas to eat at “wildlife” restaurants. Hunted or farmed wildlife are transported and kept alive at these restaurants until consumption, increasing the risk of pathogen spillover.

The wildlife trade has played a role in the emergence of severe acute respiratory syndrome (SARS), avian influenza, Ebola, and other diseases of wildlife origin [2, 9]. Guangdong Province in China was the site of the first cases of SARS in 2002. Now known to have originated in bats [10], SARS emerged in humans and other mammals in wet markets, such as Himalayan palm civets (*Paguma larvata*) and raccoon dogs (*Nyctereutes procyonoides*) [11]. The disease spread to 26 countries and infected over 8,000 people; killing nearly 800 [12]. SARS shut down trade of domestic animals and resulted in nearly $40 billion in losses to the global economy [13]. H5N1 outbreaks during 1999–2008 and H7N9 in 2013 have demonstrated the realities of the risk of disease transmission and spread in China and the detriments to animal and human health, as well as human livelihoods [9, 14]. These epidemics shed new light on how quickly and how far newly emerged zoonotic diseases can spread in today’s world. These outbreaks continue to challenge scientists, policymakers, and public health systems, exemplifying the need for a better understanding of the factors leading to emergence, and clarity regarding emerging infectious disease policy, particularly as it concerns high-risk human-animal interfaces.

Although mounting evidence implicates illegal trade and behavioral and cultural factors as influencing disease emergence, it is clear that more research is needed to identify these drivers [2, 4, 6, 7, 15]. A key aspect of understanding human-wildlife interfaces is the characterization of risk behaviors and perceptions of individuals who have wildlife contact. Specifically, there is a need to evaluate whether particular behaviors are associated with increased risk of transmission. As well, serological studies may be helpful to evaluate exposure, either current or prior, to zoonotic agents. For example, when Guan *et al*. (2003) surveyed animals in markets for evidence of SARS-like coronavirus [11], they also performed serological analysis on market employees. Seroprevalence in their study ranged widely between traders (wild-animal to vegetable), butchers and community controls.

The present study focuses on the potential for zoonotic viral transfer through contact with wildlife in Guangdong prefectures in China, and seeks to augment our understanding and identification of risky populations, occupations, and behaviors, as well as the perceptions of risk at these interfaces. We performed a serological survey and concurrent behavioral questionnaire of individuals with wildlife contact in Guangdong Province, China, in order to better characterize occupations and community-level behavioral risks that contribute to zoonotic transmission of various wildlife pathogens with pandemic potential.

## Materials and methods

### Study location and targeted population

This study took place in Guangdong Province (23°24’N, 113°30’E), China from 2009–2013. Guangdong is located on the South China Sea coast, bordering Guangxi Province, Hunan Province, Jiangxi Province and Fujian Province. It neighbors Hong Kong and Macau, and has over 50 million residents living in 9 cities: Guangzhou, Shenzhen, Dongguan, Foshan, Zhongshan, Zhuhai, Jiangmen, Huizhou and Zhaoqing. Enormous consumption of animal and animal products in this region is believed to serve an economical incentive for butchers, cooks and hunters in less developed Guangdong prefectures, e.g. Qingyuan, Yunfu and Meizhou. Guangdong Province has consistently reported the largest population in China since 2005, with the highest GDP rankings for the last 25 years. Its economy is highly dependent on the Pearl River Delta Region, which contributes around 80.0% total provincial GDP while supporting 29.0% of the provincial population.

**Fig 1:**
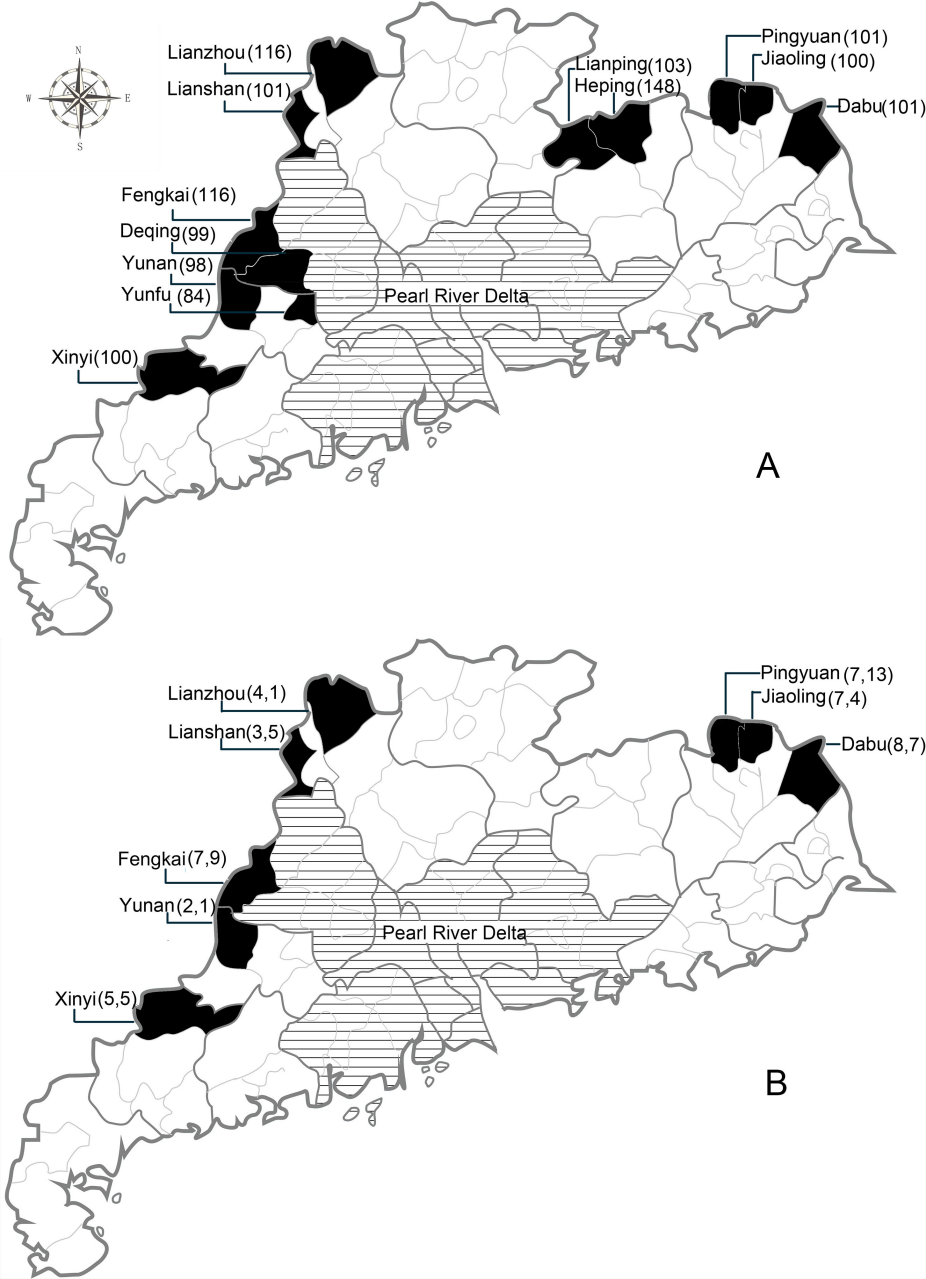
Prefecture maps of Guangdong Province, China. A) 1,267 participants were enrolled at Dabu, Jiaoling, Pingyuan, Lianping, Heping, Lianshan, Lianzhou, Yunfu, Yunan, Xinyi, Deqing and Fengkai (areas colored black). Number of respondents at each prefecture is bracketed; (B) In the brackets, in total 43 (left) seropositive respondents and 45 (right) close contacts of theirs were enrolled in the follow-up phase at Xinyi, Yunan, Fengkai, Lianshan, Lianzhou, Pingyuan, Jiaoling and Dabu (areas colored black). Together, 88 individuals participated in the follow up phase.

**Fig 2:**
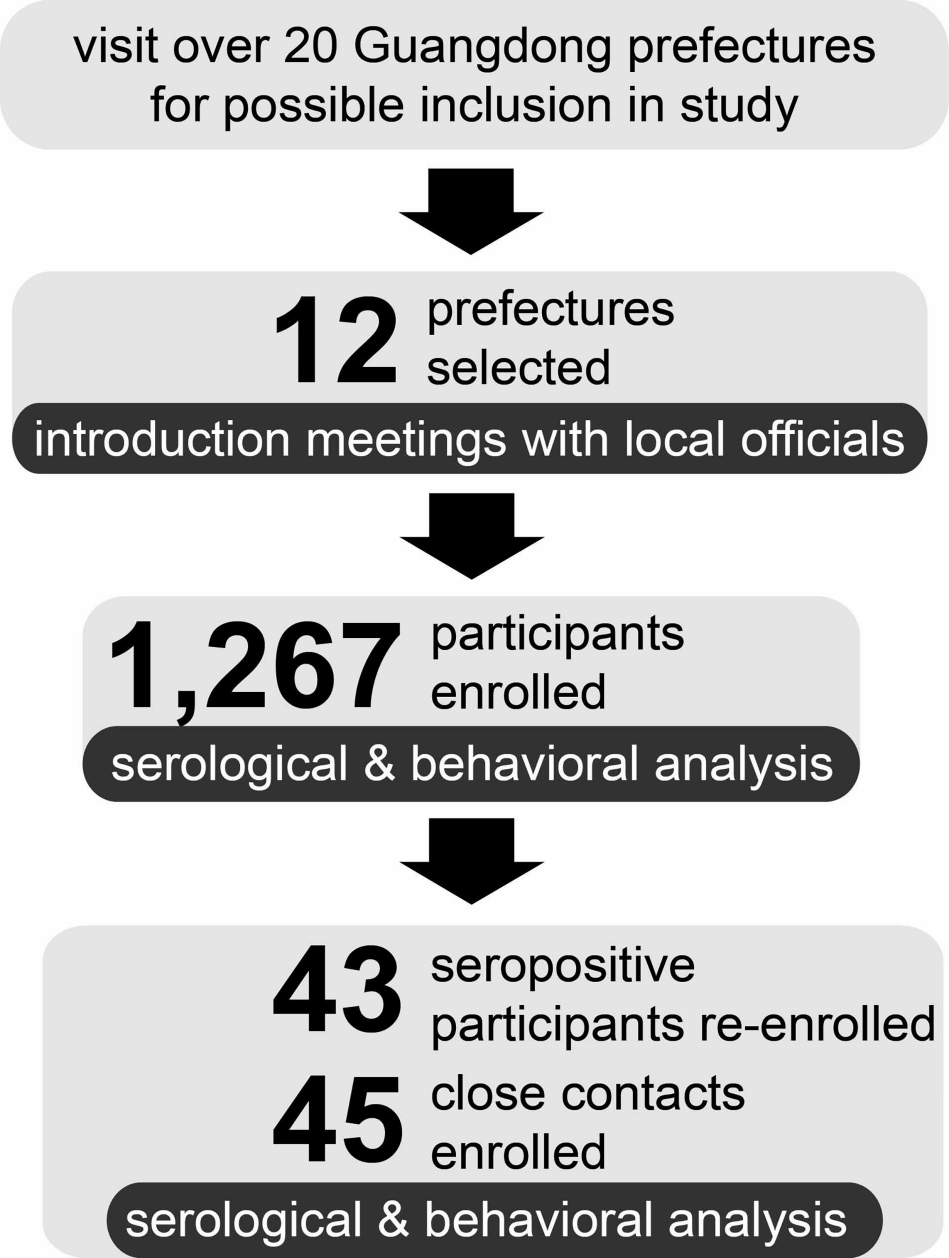
Flow diagram of recruitment and enrollment. 12 prefectures were selected after primary investigation by visiting over 20 prefectures. Totally 1,267 individuals were enrolled, and serological tests and behavioral interviews were conducted. In the follow up study, 43 respondents showing seropositive results to SARS virus, hantavirus, and/or bunyavirus in the enrollment phase and 45 of their close contacts were enrolled. Additionally, 52 respondents showing indeterminate or positive results were excluded in the follow up study since they moved to other locations between the two study phases. Another round of serological analysis and behavioral investigation were conducted to 88 respondents in the follow up study.

The study was conducted in 12 prefectures within the Guangdong Province: Dabu, Jiaoling, Pingyuan, Lianping, Heping, Lianshan, Lianzhou, Yunfu, Yunan, Xinyi, Deqing and Fengkai (Fig 1). We chose field sites within the identified prefecture areas that have wet-markets, wild-animal restaurants, and other environments with a higher risk of animal-to-human pathogen transmission. These environments, particularly the markets, were deliberately chosen due to their size and character. Similar species of wildlife, both live and butchered, were present, with similar butchering methods being utilized. Populations in these environments with high-risk animal-to-human pathogen transmission activities, such as hunting, and butchering in wet-markets and wild-animal restaurants were selected. We targeted high-risk individuals, defined as individuals with high levels of exposure to wildlife (wild animal blood or bodily fluids)—primarily hunters, persons working in wet markets and restaurants that butcher wild game, who could be followed over a period of time. Inclusion criteria included participant age between 18–65, and individuals that reported previously hunting, selling, butchering, or eating wild animals. Individuals who did not provide informed consent and/or did not agree to biological sample collection were excluded. Individuals were residents of the site area so that they were available for follow up visits. More than 20 potential field sites were visited to gauge level of risk at the site (number of high-risk animal-human interfaces). Local prefecture-level Chinese Center for Disease Control (CDC) teams were asked to pre-enroll individuals who fit the inclusion criteria and would be available for follow up visits for up to three years. Based on pre-enrollment numbers and human-animal interfaces identified at each site, 12 field sites that were identified in the previously named prefectures: Dabu, Jiaoling, Pingyuan, Lianping, Heping, Lianshan, Lianzhou, Yunfu, Yunan, Xinyi, Deqing and Fengkai were chosen to participate in the study (Fig 2). Study participation was voluntary and respondents were given the equivalent of 5 USD (approximately 30 CNY) as compensation for their time. Informed consent was obtained from all enrolled participants prior to enrollment and specimen collection. In total, 1,267 participants were enrolled in the first round of the study. The prefecture-level CDC research teams were trained in all enrollment procedures (biological and behavioral questionnaire).

### Serum collection

Ten (10) mL of peripheral venous blood was collected using pre-labeled vacuum EDTA blood collection tubes. All blood samples were chilled immediately at 4°C, and transferred to participating CDC laboratories at surveillance sites within 2 hours after sampling. Upon arrival of blood samples into the laboratories, sera were isolated by 1,500 g centrifugation for 15 min. Serum was aliquoted, flash frozen in liquid nitrogen, and then stored at -80°C.

### Risk behavior questionnaire

All study participants responded to a detailed questionnaire including questions about demographics, occupation, rural vs. urban habitation, zoonotic disease risk perceptions, and contact with particular types of wildlife through hunting, butchering, or eating. The questionnaire was based on a previous study in Cameroon and is available in the S1 File [16, 17]. It was adapted in English for the study environment in China and then translated into simplified Chinese. During the training of the local CDC research teams, pretesting of the questionnaire was done to ensure the intelligibility of the questions and proper translation. Pretesting was also done with the study population during the pre-enrollment phase for the study sites. During enrollment, a trained member of the local CDC team who was fluent in the local language of the study site administered the questionnaire. In Guangdong Province, both Mandarin and Cantonese are spoken in the study sites. The questionnaire was administered in either Mandarin or Cantonese depending on the respondent.

### Follow up study

All follow up study respondents were contacted by a member of the trained research team regarding their test results. Seropositive individuals or those with indeterminate results were referred to a local clinic/hospital where they could follow up on their results. This study did not provide additional financial or medical support for respondents who tested positive. All results were kept confidential and only reported to the respondent themselves. 95 individuals who tested seropositive from the first enrollment were asked to participate in an additional follow up sampling visit. They were also asked to invite up to three of their close contacts to enroll in the follow up study. We aimed to continue behavioral information and biological sample collection towards gauging virus transmissibility. Prior to sampling, we re-consented seropositive individuals and consented their close contacts. Due to geographic reallocation of some respondents, we were unable to locate 52 individuals with indeterminate or seropositive results for inclusion in our follow up study. We successfully re-enrolled 43 seropositive respondents and 45 of their close contacts in our follow-up study (Fig 2).

### Institutional review board (IRB) approval

This study protocol was approved by Western Institutional Review Board (protocol number: 20090204, GVFI: 06-09-057-02), United States, and Ethical Committee of Center for Disease Control and Prevention of Guangdong Province (GDCDC), China. The follow-up study protocol (protocol number: 215253–7) was approved by Ethical Committee of Center for Disease Control and Prevention of Guangdong Province, China and the University of California, Davis IRB.

### Serological analysis

The serological investigation focused on detection of hantavirus, SARS CoV, and Severe Fever with Thrombocytopenia Syndrome (SFTS) bunyavirus. This study aimed to demonstrate viral transfer associated with exposure to wildlife and the virus targets for this study were selected based on prevalence and infection associated with animal/wildlife contact as well as an interest by the GDCDC [18–20]. To determine hantavirus specific antibodies in study respondents, stored human sera were tested using Hantavirus Hantaan IgG/IgM ELISA in a single well for each sample (IBL, Hamburg, Germany) [21]. Microtiter plates were pre-coated with recombinant nucleo-capsidprotein of Hantaan virus. According to the manufacturer’s instructions, control groups and experiment group sera were diluted at 1: 100 using 1×PBS, pH 7.4, and incubated at 37°C. Coated wells were washed, and then incubated with 100 μL diluted peroxidase conjugated anti-IgG enzyme at 37°C. Color development reaction was initiated by adding a chromogenic substrate, tetramethylbenzidine, followed by a 10 minute incubation at room temperature. Reaction was quenched by adding 100 μL 0.5 M H_2_SO_4_, and then measured at 450 nm to obtain its optical density.

Human sera were also tested for SARS CoV using the SARS Coronavirus IgG Diagnostic Kit (BGI, Shenzhen, China) [22–24]. Microtiter plates were pre-coated with Vero E6 cell lysate. Cells were previously infected by SARS coronavirus that was isolated during the SARS outbreak in 2002 in China. Sera preparation and indirect ELISA were conducted as described above. The average Optical Density (OD) of negative control sera plus 0.13 was used as the cut off value as suggested by the manufacturer.

We adopted recombinant coating antigen in the diagnosis of SFTS bunyavirus using indirect ELISA. Gene encoding 6×His tagged SFTS bunyavirus HB29 nucleocapsid (N) protein was expressed in E. coli, and purified using nickel affinity chromatography. Recombinant anti-SFTSV IgG was immobilized in 96-well plate as coating antigen. Sera were diluted at 1: 100 using 1×PBS, pH 7.4, and incubated at 37°C. We used horseradish peroxidase (HRP)-conjugated anti-human IgG antibody (Sigma, Saint Louis, MO) as the secondary antibody for color development. OD was measured at 450 nm, with a reference wavelength of 620 nm. Cut-off value was determined by adding 3× SD to the mean of OD values resulting from analyses of negative control sera. Moreover, a microneutralization assay was performed to detect neutralizing antibodies against SFTS bunyavirus [25]. This was done to ensure the highest level of certainty given the newness of the SFTS virus assays. Fifty (50) μL serially diluted human sera were mixed with an equal volume of 100 TCID50 /0.1ml of SFTS bunyavirus HB29, and then incubated at 37°C for 1.5 h. Incubated mixture was added to cultured Vero cells in a 96-well plate in quadruplicate. Subsequently, the plates were incubated at 37°C with 5.0% CO_2_ for 7 d. Viral infection was assessed via an immunofluorescence assay using mouse polyclonal anti-SFTS bunyavirus antibody. End-point titer was then expressed as the reciprocal of the highest dilution of serum with viral infection prevention capability.

### Risk factor questionnaire analysis

Laboratory results and behavioral data from the questionnaire were manually entered twice (double entry system) and compared for entry error. Hand checks were also done on 10.0% of the database to further reduce chances of human entry error. Questionnaire responses and serological test results were then analyzed and compared using SAS (version 9.4; SAS Institute Inc., Cary, NC, USA). We assigned primary occupation to the categories of hunter, agriculture worker, butcher, market worker, and restaurant worker based on questionnaire responses. We also aggregated reported behaviors to assign an “ever” status to each individual in terms of eating, butchering, or hunting wildlife. Basic descriptive univariate statistics were carried out on the data set. We then examined bivariate associations between serological status for the three viruses of interest and independent demographic and risk behavior variables. Variables showing bivariate association with seropositivity at a p value of 0.1 or less were included in multivariate logistic models for seropositivity to individual viruses or any of the three viruses using Firth’s Penalized Likelihood adjustment to address small sample size and possible collinearity [26].

Since butchers were the only occupational group showing increased risk of seropositivity, we also performed a subanalysis of specific behaviors performed by butchers, again including behaviors with bivariate association of p = 0.1 or less in a multivariate logistic model.

## Results

### Demographics

A total of 1,267 individuals provided a baseline blood sample for serological testing and completed a behavioral risk factor survey. The demographics of these individuals are shown in Table 1.

**Table 1:** Demographics of the study population.

Variable	Total (%) N = 1,267
**Gender**	
Female	232 (18)
Male	985 (78)
No answer	50 (4)
**Age (y)**	36.97 ± 11.03
**Education level**	
Primary school	219 (17)
Junior school	791 (62)
Senior school	177 (14)
University	18 (1)
Apprenticeship	49 (4)
None	13 (1)

The study population was predominantly younger than age 60, with a mean age of 36.97 ±11.03 years. Junior high school was most frequently reported as the highest level of education attained. Most of the respondents were married or living with their partner and had between 1–2 children, with a total of 3–6 people living in the house.

Traveling to a forest at least once a month, which may provide additional exposure to wild animals, was reported by 67.0% of respondents (data not shown). Some of the most common reasons for traveling to the forest were fieldwork (56.0%), gathering of fruit and vegetables (21.0%), collecting firewood (11.0%), and hunting (10.0%). More respondents reported ever living in a city than not, with the most common urban occupations being restaurant work followed by “other” and factory jobs.

Table 2 describes type and location of occupational activities. Restaurant-related occupations such as cook or kitchen worker were the most commonly reported primary occupation, followed by agriculture, butcher, and jobs working in a market.

**Table 2:** Primary occupation and urban residence.

Variable	Total (%)* N = 1,267
**Primary occupation**	
Cook /Other restaurant worker	463 (37)
Agriculture	220 (17)
Butcher	111 (9)
Market worker	92 (7)
Housework	62 (5)
Hunting	38 (3)
Other	145 (11)
No Answer	135 (11)
**Ever lived in the city**	
No	442 (35)
Yes	825 (65)

* Numbers add up to more than 100% because the respondent had the option to select multiple occupations

Table 3 summarizes reported exposures to wild animals. Almost all respondents indicated that they had ever touched any live or dead wild animal. Having butchered and eaten wild animals were the most common types of animal interactions, and injury was experienced by less than a quarter of the respondents.

**Table 3:** Reported exposures to wild animals.

Variable	Total (%) N = 1,267
**Hunted any wild animal**	
No	1,043 (82)
Yes	224 (18)
**Butchered any wild animal**	
No	301 (24)
Yes	966 (76)
**Eaten any wild animal**	
No	419 (33)
Yes	848 (72)

Table 4 shows the beliefs and reported protective behavior regarding contact with animals and zoonotic disease transmission. Less than one third of respondents reported believing that they could get infected from contact with blood or other types of contact with wild animals. A smaller proportion reported taking at least one type of protective measure to prevent infection in such circumstances. Among those who reported taking protective measures, use of gloves, and avoiding touching the animal were the most commonly reported protective practices.

**Table 4:** Beliefs and reported behaviors.

Variable	Total (%) N = 1,267
**Believe one can get infection from animals/blood**	
No	594 (47)
Yes	402 (32)
Don’t Know/Refused	271 (21)
**Take Any type of protective measure**	
None	810 (64)
At least one type	218 (17)
Don’t Know	239 (19)
If yes, type of protective measure (n = 218)	
Gloves	128 (59)
Avoidance of exposure	42 (19)
Other	48 (22)

### Serological results

#### Risk factors for seropositivity

Table 5 summarizes the results of baseline testing of blood for evidence of antibodies against zoonotic wildlife viruses. The overall rate of seropositivity to any of the other three tested pathogens (Hantavirus, SARS CoV, or SFTS bunyavirus) was low among the study population (approximately 4%). Seropositivity in respondents was only found in the first phase of enrollment. Among contacts of these seropositive respondents (n = 45), all individuals were seronegative for the pathogens of interest. The most common seropositivity was for Hantavirus (2.3% of the study population). However, among the 112 persons reporting that their occupation was a butcher, the rate of seropositivity to at least one pathogen was higher (9.0%), and this elevated risk was statistically significant (Fisher’s Exact Test, p = 0.005). Butchers had elevated seropositivity rates to all three of the viruses compared to the overall study population. No other occupational group showed an increased risk of seropositivity. In a multivariate model of the butchers, those who reported butchering porcupines showed an elevated risk of seropositivity (data not shown). There was no increased risk among the butchers associated with not wearing gloves or taking fewer protective measures.

**Table 5:** Serological results, by primary occupation and reported activities.

Risk Factor	SARS Total Number (%)	Hanta Total Number (%)	Bunya Total Number (%)	Any Total Number (%)
**Primary Occupation**				
Butcher (n = 112)	3 (2.7%) p = 0.18	5 (4.5%) p = 0.17	2 (1.8) p = 0.18	10 (9.0%) p = 0.005
Hunter (n = 38)	0 (0.0%)	0 (0.0%) p = 0.34	0 (0.0%) p = 0.9	0 (0.0%) p = 0.19
Cook or Restaurant (n = 463)	5 (1.1%) p = 0.62	11 (2.4%) p = 0.88	0 (0%) p = 0.62	16 (3.5%) p = 0.37
Agriculture (n = 220)	4 (1.8%) p = 0.52	3 (1.4%) p = 0.31	2 (0.9%) p = 0.52	9 (4.1%) p = 0.98
Housework (n = 62)	0 (0%) p = 0.9	1 (1.6%) p = 0.71	0 (0%) p = 0.9	1 (1.6%) p = 0.31
Market (n = 92)	0 (0%) p = 0.6	3 (3.3%) p = 0.51	1 (1.1%) p = 0.63	3 (3.3) p = 0.67
All (n = 1267)	17 (1.3%)	29 (2.3)	7 (0.6%)	53 (4.2%)
**Specific Behavior**				
Ever hunting (n = 224)	1 (0.4) p = 0.33	0 p = 0.01	2 (0.9) p = 0.33	3 (1.3%) p = 0.02
Ever butchering (n = 966)	14 (1.4) p = 0.77	26 (2.7) p = 0.09	6 (0.6) p = 0.77	46 (4.8%) p = 0.07
Ever eaten wild animals (n = 848)	12 (1.4) p = 0.74	18 (2.1) p = 0.57	7 (0.8) p = 0.9	36 (4.3%) p = 0.72

By contrast, the 38 persons reporting hunting as a profession showed a decreased risk for seropositivity as did those reporting their profession as housework. No other occupational groups showed significantly increased or decreased risk. Persons who reported ever hunting also had a lower risk of seropositivity compared to those who never hunted. Age and gender did not show a significant association with seropositivity, nor did reporting living in a city or going frequently into the forest. While 111 persons reported butchering as a profession, a larger number (966) reported ever butchering a wild animal; this was associated with an elevated risk of seropositivity, however when the professional butchers were removed from this group, the risk was no longer significant. As shown in the Table S1 Table, reported contact with certain wildlife species such as civets (for Hanta and Bunyavirus) and field rats (for SARS) showed elevated risk for certain viruses in bivariate analyses.

In multivariate models of specific wildlife contact, (Table 6), the principal association remaining significant was eating civets (associated with Hantavirus seropositivity). The risk estimate for Bunyavirus was elevated but did not reach statistical significance.

**Table 6:** Multivariate models for seropositivity–common activities, animal specific.

	Any Seropositive		Hanta		SARS		Bunya	
	OR	95% CI	OR	95% CI	OR	95% CI	OR	95% CI
**Boar-Eat**	-	-	-	-	-	-	1.5	0.3–8.7
**Boar-Hunt**	0.3	0.01–5.2	-	-	-	-	-	-
**Civet-Eat**	1.8	0.8–4.0	3.5	1.4–8.5	-	-	4.2	0.9–19.4
**Field rat-Eat**	2.3	0.99–5.4	-	-	1.1	0.2–8.2	-	-
**Field rat-Butcher**	-	-	-	-	2.3	0.3–16.9	-	-
**Rat-Eat**	-	-	-	-	2.8	0.7–10.7	-	-
**Rat-Butcher**	-	-	-	-	0.99	0.2–4.4	-	-
**Wildbird-Eat**	-	-	-	-	-	-	2.6	0.5–13.5
**Wildbird-Butcher**	1.7	0.96–2.9	-	-	-	-	-	-
**Wildbird-Hunt**	0.3	0.06–1.6	0.1	0.007–1.9	-	-	-	-

## Discussion

This study, examining the association between reported wildlife contact and seropositivity for wildlife zoonotic viruses, found detectable levels of antibodies for several pathogens in the population surveyed. Overall, the population had contact with multiple species of animals. Among reported primary occupations, working as a butcher was associated with an increased risk of infection with wildlife pathogens, while professional hunters were at decreased risk, and no increased risk was seen for market or restaurant workers. Contact with certain animals, such as civets and field rats, showed evidence of increased risk of seropositivity. Few people in the study reported using protective measures such as gloves when working with wild animals, and less than a third of individuals reported believing that they could become infected through wildlife contact. When seropositive individuals were followed over time, no evidence of infection of close contacts was seen, although the number of contacts enrolled was small and there are limited conclusions that can be draw about alternative transmissions risks. Further investigation is warranted to gauge transmissibility of the viruses studied.

These findings have a number of implications for prevention of zoonotic disease transmission and outbreaks. Those individuals at the animal-human interface (such as persons butchering wild animals) that are highly exposed to wild animals are influential in disease amplification and dissemination and reduction of risk factors can play a large role in mediating potential outbreaks.

While we identified butchers as an occupational group at increased risk, better understanding of the specific exposures driving this increased risk would require more in-depth study. Among butchers, we did find an elevated risk of virus seropositivity associated with butchering porcupines, but due to small numbers, such a finding must be considered preliminary. This study also identified an increased risk of seropositivity with contact with civets and field rats which could be due to multiple factors. Civets and field rats are among the most popular types of wild animals found in markets and restaurants and prolific contact may result in higher risk. There is also a bias related to the viruses that the study targeted with two of the three (SARS CoV and hantavirus) associated with both civets and rats.

This study had a number of limitations, including selection bias. We deliberately targeted high risk individuals, making our sample less representative of the general population. Conversely, it is possible that there could be differential participation rates between high risk and lower risk individuals, further limiting any extrapolation to community levels. We focused on wildlife contact, and therefore were unable to assess the risk impact of contact with other animal species including livestock and companion animals. Further research should aim to include larger sample sizes from more geographically diverse regions, and assess seroprevalence rates in random population samples. Underreporting of risk activities on the behavioral survey may have occurred, since many activities related to wild animals are illegal in China. In addition, since this was a hypothesis generating study, the analysis involved multiple comparisons. Therefore, any reported association should be viewed with caution. While this study involved serological testing for more than one potential wildlife pathogen, there are numerous other wildlife pathogens, including bacterial and viral agents, which the study did not investigate. It is therefore possible that this study failed to detect other significant transmission events of human health relevance. Future studies should consider such possibilities.

The finding that less than one third of the surveyed population, most of whom reported wild animal contact, believed that they could become infected through such contact, indicates the need for targeted educational programs for prevention, especially among those involved in butchering wildlife for consumption. Targeted education programs should aim to increase knowledge of disease risk, perception of risk, and risky behaviors in the identified high-risk sub groups.

Previous studies indicate the need to target the high-risk sub group of hunters [16, 17]. In this study, however, hunters and persons reporting ever hunting or hunting as a profession exhibited a lower rate of seropositivity compared with the overall population. It is possible that hunting activities may be underreported, but the results of this study indicate some of the difficulties of relying on self report to identify individuals at increased risk.

Due to the huge demand by the local residents and immigrants in the larger Pearl River Delta area, which includes Guangdong, Hong Kong and Macau, increasing number of individuals engaged in animal production and value chain systems are believed to continue to come into contact with wild animals and engage in risky behaviors increasing the risk of a possible infectious disease outbreak that could have a potentially severe impact. Wild animals are an important part of Southern Chinese economy and culture, and pragmatic policy changes should be implemented to control the risks of possible disease outbreaks. These policy changes should aim to increase risk perception and uptake of precautionary behaviors without risking the cultural and financial security of the community. Results of the analyses of these research findings can be used to inform public health strategies such as communication and educational campaigns, targeting those behaviors that place individuals at higher risk of disease.

Our study provides evidence towards recommendations that include: an increase in the level of collaboration between animal and human health programs targeting human-animal interfaces to increase efforts to control and prevent outbreaks; development of new educational campaigns to increase knowledge and awareness of infectious diseases that target specific sub-groups, such as butchers, that are at increased risk; and increase monitoring of high-risk groups to detect continued transmission of zoonotic diseases in a timely manner.

## Supporting information

S1 File Study questionnaire for persons hunting, butchering, eating and/or keeping wild animals as pets. (PDF)

S1 Table Prevalence of seropositivity for tested viruses, by reported wildlife exposure. (PDF)
