# Correction: Serologic and behavioral risk survey of workers with wildlife contact in China

**DOI:** 10.1371/journal.pone.0196701

**Published:** 2018-04-30

**Authors:** Corina Monagin, Blanca Paccha, Ning Liang, Sally Trufan, Huiqiong Zhou, De Wu, Bradley S. Schneider, Aleksei Chmura, Jonathan H. Epstein, Peter Daszak, Changwen Ke, Peter M. Rabinowitz

The ninth author’s middle initial is missing. The correct name is: Jonathan H. Epstein.
