# Supplementary material for: Serologic and behavioral risk survey of workers with wildlife contact in China
Source: PLoS One. 2018 Apr 3;13(4):e0194647. doi: 10.1371/journal.pone.0194647 (PMC5882129; doi:10.1371/journal.pone.0194647)
Supplement: S1 File — (PDF) [file pone.0194647.s001.pdf]

Attachment F3

STUDY QUESTIONNAIRE FOR PERSONS HUNTING, BUTCHERING, EATING, AND/OR KEEPING WILD ANIMALS AS PETS:

Participant's Name (Please print)____
Last First Middle Initial

Participant ID Number: ____

Interview Date:___ ___ / ___ ___ / ___ ___
Month / Day / Year

City, Country, Site Code: ____

Telephone Number (if available): (_________) _________ - ________________

Address:
____
____
____

Date of Birth: ___ ___ / ___ ___ / ___ ___
Month / Day / Year

THIS PAGE WILL BE SEPARATED FROM THE QUESTIONNAIRE AND STORED LOCALLY TO PROTECT STUDY PARTICIPANT'S CONFIDENTIALITY.

DATE ___ ___ / ___ ___ / ___ ___ PARTICIPANT ID NUMBER _________________
month / day / year

BODY TEMP: ___________________

SECTION A

BACKGROUND CHARACTERISTICS

A1	How old are you?	AGE IN YEARS	A1
		REFUSED 888	
		UNKNOWN 999	
A2	RESPONDANT GENDER	MALE 1	A2
		FEMALE 2	

A3.1	DISTRICT LOCATION	DISTRICT NAME: ________________________________	
A3.2	COMMUNITY LOCATION	COMMUNITY NAME: _____________________________	A3
A4	What is your ethnic group?	ETHNIC GROUP: _________________________________<br><br>REFUSED 888<br>UNKNOWN 999	A4
A6	Do you own any of the following items?<br><br>*MARK ALL THAT APPLY*	TELEVISION 1<br>CAR/TRUCK 2<br>BICYCLE 3<br>SEWING MACHINE 4<br>MOTORCYCLE 5<br><br>REFUSED 888<br>UNKNOWN 999	A6
A7	What is your highest education level?	NONE 1<br>PRIMARY SCHOOL 2<br>JUNIOR SCHOOL 3<br>SENIOR SCHOOL 4<br>APPRENTICESHIP 5<br>UNIVERSITY 6<br><br>REFUSED 888<br>UNKNOWN 999	A7
A8	How many times have you traveled to a capital city?	NEVER 1<br>1-2 TIMES 2<br>3-10 TIMES 3<br>MORE THAN 10 TIMES 4<br><br>REFUSED 888<br>UNKNOWN 999	A8

A9	How often do you go to the fields/forest around your village?<br><br>*IF NEVER, SKIP TO QUESTION A12*	NEVER 1<br>ONCE A MONTH 2<br>TWICE TO FOUR TIMES A MONTH 3<br>MORE THAN FOUR TIMES A MONTH 4<br><br>REFUSED 888<br>UNKNOWN 999	>Q A12	A9
A10	What is the purpose of this travel?<br><br>*CHECK ALL THAT APPLY*<br><br>*IF OTHER, PLEASE SPECIFY*	HUNTING 1<br>COLLECTING WATER 2<br>GATHERING FRUIT/VEGETABLES 3<br>COLLECTING FIREWOOD 4<br>FISHING 5<br>FIELD WORK 6<br>SPECIFY: __________________OTHER 7<br><br>REFUSED 888<br>UNKNOWN 999		A10
A11	What kind of work do you do, Order up to three occupations '1', '2', '3'<br><br>*NOTE: 1=most important… 3=less important*<br><br>*IF OTHER, PLEASE SPECIFY*	AGRICULTURAL 1<br>HOUSEWORK IN OWN HOME 2<br>HUNTING 3<br>CHILD CARE OF OWN CHILDREN 4<br>FISHING 5<br>MARKET PERSON 6<br>STUDENT 7<br>SPECIFY:________________ __OTHER 8<br><br>REFUSED 888<br>UNKNOWN 999		A11
A12	Have you ever lived in a capital city?<br><br>*IF NO, SKIP TO QUESTION A14*	YES 1<br>NO 2<br><br>REFUSED 888<br>UNKNOWN 999	 >Q A14	A12

A13.1	What capital city or cities?	CITY/CITIES: ______________________<br>REFUSED 888<br>UNKNOWN 999	A13
A13.2	IF YOU LIVE IN THE CITY, WHAT IS YOUR OCCUPATION?	RESTAURANT WORKER 1<br>BUTCHER 2<br>OFFICE WORKER 3<br>FACTORY WORKER 4<br>HOUSEWIFE 5<br>SELLER 6<br>OTHER 7<br>REFUSED 888<br>UNKNOWN 999	
A14	Have you ever had a blood transfusion?	YES 1<br>NO 2<br>REFUSED 888<br>UNKNOWN 999	A14
A15	Have you ever donated blood?	YES 1<br>NO 2<br>REFUSED 888<br>UNKNOWN 999	A15
A16	Have you had a serious illness?<br>*IF NO, SKIP TO QUESTION A18*	YES 1<br>NO 2 >Q A18<br>REFUSED 888<br>UNKNOWN 999	A16
A17	If yes, please describe	MEDICAL HISTORY:<br>______________________<br>______________________	A17

			REFUSED 888<br>UNKNOWN 999		
A18	Have you ever been vaccinated for smallpox?<br><br>*IF NO, SKIP TO QUESTION A20*		YES 1<br>NO 2<br><br>REFUSED 888<br>UNKNOWN 999	 >Q A20	A18
A19	Is there a pox mark present?<br><br>*INTERVIEWER SHOULD CHECK FOR POX MARK*		YES 1<br>NO 2<br><br>REFUSED 888<br>UNKNOWN 999		A19
A20	Are there any cultural practices in your community that involve blood?<br><br>*IF NO, SKIP TO QUESTION A22*		YES 1<br>NO 2<br><br>REFUSED 888<br>UNKNOWN 999	 >Q A22	A20
A21	Please describe these cultural practices	PRACTICE:<br>________________________________<br>________________________________	 <br><br><br>REFUSED 888<br>UNKNOWN 999		A21
A22	How many children do you have?	NUMBER BORN:	REFUSED 888<br>UNKNOWN 999		A22

A23	How many people are currently living in your household?	NUMBER:<br><br>REFUSED 888<br>UNKNOWN 999		A23
A24	Have you ever been married?<br><br>*IF NO, GO ON TO SECTION B*	YES 1<br>NO 2<br><br>REFUSED 888<br>UNKNOWN 999	>SEC B	A24
A26	Has one of your partners lived in a capital city?<br><br>*IF NO, SKIP TO QUESTION A28*	YES 1<br>NO 2<br><br>REFUSED 888<br>UNKNOWN 999	>Q A28	A26
A27	What city or cities has your partner lived in?	CITY/CITIES:<br>______________________________<br>______________________________<br><br>REFUSED 888<br>UNKNOWN 999		A27
A28	What kind of work does your partner do, or what kind of activities do they do on an average day, whether they receive money for them or not? Order up to 3 occupations ‘1’, ‘2’, ‘3’<br><br>*NOTE: 1=most important… 3=less important*	AGRICULTURAL 1<br>HOUSEWORK IN OWN HOME 2<br>HUNTING 3<br>CHILD CARE OF OWN CHILDREN 4<br>FISHING 5<br>MARKET PERSON 6<br>STUDENT 7<br>SPECIFY:______________OTHER 8<br><br>REFUSED 888<br>UNKNOWN 999		A28

PLEASE SPECIFY IF OTHER

A29	What is the ethnic background of your partner?	ETHNIC GROUP: ________________________________<br><br>REFUSED 888<br>UNKNOWN 999	A29

SECTION B

WILD ANIMAL EXPOSURE QUESTIONS

B1	Have you ever touched a live or dead monkey, ape, chimp, gorilla, wild pig, wild bird, or wild animal?<br><br>*IF NO, DONE WITH QUESTIONAIRE*	YES 1<br>NO 2<br><br>REFUSED 888<br>UNKNOWN 999	 >DONE	B1

YES *(GO TO TABLE BELOW)*

B.2 *Show study participant the different species of monkey and other animal pictures. If the participant recognizes a particular animal in the picture, ask the participant to indicate the activity [hunted, butchered, kept as pet, eaten, found dead and collected, injured by or not] they have performed on the animal. For each activity also ask the participant to indicate the duration of time (in months unless otherwise noted) spent doing the activity and document this in the table.*
*Please fill with **Y**(Yes) or **N**(No) and use the codes for the coded sections as specified below: If you have filled **Y**(Yes) in a box for questions 1, 2 or 3, then indicate the number of animals per month during the dry season for which this activity was performed.*

**Number**	**1 Hunt**		**2 Butcher**		**3 Eaten**		**4 Pet (If yes, # of yrs)**		**5 Found dead & collected or eaten (Y/N)**	**6 Injury #1 (If yes, Code)**				**7 Injury #2 (If yes, Code)**				**8 Injury #3 (If yes, Code)**			
**Animal**	y/n	#/mo	y/n	#/mo	y/n	#/mo	y/n	# yrs		**code**	clean (y/n)	med (y/n)	year	**code**	clean (y/n)	med (y/n)	year	**code**	clean (y/n)	med (y/n)	year
**Bat**																					
**Wild boar/pig**																					
**Rabbit**																					
**Hedgehog**																					
**Rat**																					
**Wild Bird**																					
**Dog**																					
**Badger**																					
**Weasel**																					
**Cat**																					

**Civit**																					
**Number**	**1 Hunt**		**2 Butcher**		**3 Eaten**		**4 Pet (If yes, # of yrs)**		**5 Found dead & collected or eaten (Y/N)**	**6 Injury #1 (If yes, Code)**				**7 Injury #2 (If yes, Code)**				**8 Injury #3 (If yes, Code)**			
**Animal**	y/n	#/mo	y/n	#/mo	y/n	#/mo	y/n	# yrs		**code**	clean (y/n)	med (y/n)	year	**code**	clean (y/n)	med (y/n)	year	**code**	clean (y/n)	med (y/n)	year
**Lynx**																					
**Mongoose**																					
**Leopard Cat**																					
**Anteater**																					
**Squirrel**																					
**Deer**																					
**Muntjac (small deer)**																					
**Bear**																					
**Loris**																					
Fill in others as needed																					

**Number**	**1 Hunt**		**2 Butcher**		**3 Eaten**		**4 Pet (If yes, # of yrs)**		**5 Found dead & collected or eaten (Y/N)**	**6 Injury #1 (If yes, Code)**				**7 Injury #2 (If yes, Code)**				**8 Injury #3 (If yes, Code)**			
**Animal**	y/n	#/mo	y/n	#/mo	y/n	#/mo	y/n	# yrs		**code**	clean (y/n)	med (y/n)	year	**code**	clean (y/n)	med (y/n)	year	**code**	clean (y/n)	med (y/n)	year

<u>Codes for Injury Type:</u>

<u>HUNT</u>

(HB) Bite
(HS) Scratch
(HWS) Wound due to wire snares
(HM) Wound due to machete
(HO Any other injury, please specify

<u>BUTCHER</u>

(BB) Bite

(BS) Scratch
(BO) Any other injury, please specify

PET
(PB) Bite
(PS) Scratch
(PO) Any other injury, please specify

B3	Do you think you can get infections from animals/animal blood?<br><br>*IF NO, SKIP TO SECTION C*	YES 1<br>NO 2<br><br>REFUSED 888<br>UNKNOWN 999	 >SEC C	B3
B4	What do you do to protect yourself?	PROTECTIVE MEASURES:<br>____________________<br><br>REFUSED 888<br>UNKNOWN 999		B4

SECTION C

FOR HUNTERS ONLY

C1	What methods do you use to capture these animals?<br><br>*CIRCLE ALL THAT APPLY*<br><br>*IF OTHER, PLEASE SPECIFY*	WIRE SNARE 1<br>BOW 2<br>HANDS 3<br>GUN 4<br>MACHETE 5<br>TRADITIONAL SNARE 6<br>NET 7<br>SPECIFY:________________OTHER 8<br><br>REFUSED 888<br>UNKNOWN 999	C1
C2	Did you have any wounds while you captured these animals?	YES 1<br>NO 2<br><br>REFUSED 888<br>UNKNOWN 999	

SECTION D

FOR BUTCHERS ONLY

D1	What method or methods do you use to butcher the animal(s)?<br><br>*CIRCILE ALL THAT APPLY*<br><br>*IF OTHER, PLEASE SPECIFY*	MACHETE 1<br>BARE HANDS 2<br>WEAR GLOVES 3<br>SPECIFY:________________OTHER 4<br><br>REFUSED 888<br>UNKNOWN 999	D1

D2	Did you have any wounds while you butchered these animals?	YES 1<br>NO 2<br><br>REFUSED 888<br>UNKNOWN 999		

SECTION E

FOR PET OWNERS ONLY

E1	Did you ever have a pet animal that died?<br><br>*IF NO, SKIP TO QUESTION E3*	YES 1<br>NO 2<br><br>REFUSED 888<br>UNKNOWN 999	 >Q E3	E1
E2	How did this pet die?	METHOD OF DEATH:<br>________________________________<br><br>REFUSED 888<br>UNKNOWN 999		E2
E3	What species of animal was this pet?	DOG 1<br>CAT 2<br>BIRD 3<br>REPTILE 4<br>PIG 5<br>RODENT 6<br>OTHER__________________________7<br><br>REFUSED 888<br>UNKNOWN 999		

SECTION F

CONSUMERS OF BUSHMEAT ONLY

F1	Have you ever eaten uncooked animal meat, blood, or organs?<br><br>*IF NO, DONE WITH QUESTIONAIRE*	YES 1<br>NO 2<br><br>REFUSED 888<br>UNKNOWN 999	 >DONE	F1
F2	What kind of animals have you eaten uncooked?	KINDS EATEN:<br>________________________________<br>________________________________<br><br>REFUSED 888<br>UNKNOWN 999		F2
F3	What is your favorite part of the animal to eat?	FAVORITE PART EATEN:<br>________________________________<br>________________________________		F3

REFUSED 888
UNKNOWN 999

END

*Thank you for participating in this study. (*Please return this completed questionnaire to the Project Coordinator.)
