# Supplementary material for: Serologic and behavioral risk survey of workers with wildlife contact in China
Source: PLoS One. 2018 Apr 3;13(4):e0194647. doi: 10.1371/journal.pone.0194647 (PMC5882129; doi:10.1371/journal.pone.0194647)
Supplement: S1 Table — (PDF) [file pone.0194647.s002.pdf]

S1 Table: Prevalence of seropositivity for tested viruses, by reported wildlife exposure.

Seropositivity (n=1267)	% Any Sero Neg (n=1214)	% Any Sero Pos (n=53)	% Hanta Neg (n=1238)	% Hanta Pos (n=29)	% SARS Neg (n=1250)	% SARS Pos (n=17)	% Bunya Neg (n=1260)	% Bunya Pos (n=7)
**Anteater-Butcher**	0.2	0.0	0.2	0.0	0.2	0.0	0.2	0.0
**Anteater-Eat**	0.2	0.0	0.2	0.0	0.2	0.0	0.2	0.0
**Badger-Butcher**	1.6	0.0	1.6	0.0	1.6	0.0	1.6	0.0
**Badger-Eat**	1.2	0.0	1.1	0.0	1.1	0.0	1.1	0.0
**Badger-Hunt**	0.7	0.0	0.6	0.0	0.6	0.0	0.6	0.0
**Bat-Butcher**	2.6	1.9	2.5	3.4	2.6	0.0	2.5	0.0
**Bat-Eat**	2.4	0.0	2.3	0.0	2.3	0.0	2.3	0.0
**Bat-Hunt**	1.6	0.0	1.5	0.0	1.5	0.0	1.5	0.0
**Bear-Butcher**	0.3	0.0	0.3	0.0	0.3	0.0	0.3	0.0
**Bear-Eat**	0.2	0.0	0.2	0.0	0.2	0.0	0.2	0.0
**Boar-Butcher**	26.4	28.3	26.3	34.5	26.6	11.8	26.3	42.8
**Boar-Eat**	36.4	41.5	36.4	44.8	36.7	29.4	36.4	71.4*
**Boar-Hunt**	7.1	0**	6.9	0.0	6.9	0.0	6.8	0.0
**Cat-Butcher**	18.5	22.6	18.7	17.2	18.5	29.4	18.5	42.9
**Cat-Eat**	20.3	15.1	20.1	20.7	20.3	5.9	20.1	28.6
**Cat-Hunt**	1.9	0.0	1.9	0.0	1.8	0.0	1.8	0.0
**Civet-Butcher**	11.0	11.3	11.0	13.8	11.1	5.9	11.0	28.6
**Civet-Eat**	8.2	15.1*	8.2	20.7**	8.6	0.0	8.3	42.9**
**Civet-Hunt**	4.4	0.0	4.4	0.0	4.3	0.0	4.3	0.0
**Cynomolgus-Butcher**	0.2	0.0	0.2	0.0	0.2	0.0	0.2	0.0

**Cynomolgus-Eat**	0.2	0.0	0.2	0.0	0.2	0.0	0.2	0.0
**Deer-Butcher**	2.6	5.7	2.7	3.4	2.7	5.9	2.7	14.3
**Deer-Eat**	3.4	5.7	3.5	3.4	3.4	5.9	3.4	14.3
**Deer-Hunt**	0.3	0.0	0.3	0.0	0.3	0.0	0.3	0.0
**Dog-Butcher**	37.6	41.5	37.7	37.9	37.5	52.9	37.7	42.9
**Dog-Eat**	50.0	52.8	50.1	51.7	50.1	52.9	50.0	71.4
**Dog-Hunt**	3.6	0.0	3.6	0.0	3.5	0.0	3.5	0.0
**Field rat-Butcher**	5.8	9.4	6.1	3.4	5.8	17.6*	6.0	14.3
**Field rat-Eat**	6.3	13.2*	6.5	10.3	6.4	17.7*	6.5	14.3
**Field rat-Hunt**	2.9	3.8	3.0	0.0	2.9	5.9	2.9	14.3
**Hedge-Butcher**	3.0	5.7	3.0	6.9	3.1	0.0	3.0	14.3
**Hedge-Eat**	2.7	3.8	2.7	3.4	2.8	0.0	2.7	14.3
**Hedge-Hunt**	1.1	0.0	1.1	0.0	1.0	0.0	1.0	0.0
**Leopard-Butcher**	0.7	0.0	0.7	0.0	0.7	0.0	0.7	0.0
**Leopard-Eat**	0.3	0.0	0.3	0.0	0.3	0.0	0.3	0.0
**Leopard-Hunt**	0.1	0.0	0.1	0.0	0.1	0.0	0.1	0.0
**Loris-Butcher**	0.2	0.0	0.2	0.0	0.2	0.0	0.2	0.0
**Loris-Eat**	0.2	0.0	0.2	0.0	0.2	0.0	0.2	0.0
**Lutra-Butcher**	0.7	0.0	0.7	0.0	0.7	0.0	0.7	0.0
**Lutra-Eat**	0.7	0.0	0.7	0.0	0.7	0.0	0.7	0.0
**Lutra-Hunt**	0.1	0.0	0.1	0.0	0.1	0.0	0.1	0.0
**Lynx-Butcher**	0.4	0.0	0.4	0.0	0.4	0.0	0.4	0.0

**Lynx-Eat**	0.4	0.0	0.4	0.0	0.4	0.0	0.4	0.0
**Lynx-Hunt**	0.3	0.0	0.3	0.0	0.3	0.0	0.3	0.0
**Mongoose-Butcher**	0.6	0.0	0.6	0.0	0.6	0.0	0.6	0.0
**Mongoose-Eat**	0.2	0.0	0.2	0.0	0.2	0.0	0.2	0.0
**Muntjac-Butcher**	7.2	5.7	7.1	6.9	7.2	0.0	7.1	14.3
**Muntjac-Eat**	6.6	3.8	6.5	3.4	6.6	0.0	6.4	14.3
**Muntjac-Hunt**	2.8	0.0	2.7	0.0	2.7	0.0	2.7	0.0
**Pangolin-Butcher**	5.4	5.7	5.3	6.9	5.4	5.9	5.4	0.0
**Pangolin-Eat**	6.0	3.8	6.0	3.4	5.9	5.9	6.0	0.0
**Pangolin-Hunt**	2.6	0.0	2.5	0.0	2.5	0.0	2.5	0.0
**Porcupine-Butcher**	5.7	11.3	5.8	10.3	5.8	11.8	5.9	14.3
**Porcupine-Eat**	6.7	7.5	6.8	3.4	6.6	11.8	6.7	14.3
**Porcupine-Hunt**	2.3	0.0	2.3	0.0	2.2	0.0	2.2	0.0
**Rabbit-Butcher**	27.3	30.2	27.2	37.9	27.6	17.6	27.5	28.6
**Rabbit-Eat**	21.0	20.8	20.8	27.6	21.2	5.9	20.9	42.9
**Rabbit-Hunt**	5.2	0.0	5.1	0.0	5.0	0.0	5.0	0.0
**Rat-Butcher**	13.9	17.0	14.1	10.3	13.8	29.4*	14.0	14.3
**Rat-Eat**	14.9	17.0	15.2	6.9	14.7	35.3**	15.0	14.3
**Rat-Hunt**	4.4	1.9	4.4	0.0	4.3	0.0	4.2	14.3
**Squirrel-Butcher**	1.7	0.0	1.7	0.0	1.7	0.0	1.7	0.0
**Squirrel-Eat**	2.5	0.0	2.4	0.0	2.4	0.0	2.4	0.0

**Squirrel-Hunt**	4.1	0.0	4.0	0.0	4.0	0.0	4.0	0.0
**Weasel-Butcher**	2.7	3.8	2.7	6.9	2.8	0.0	2.8	0.0
**Weasel-Eat**	2.2	1.9	2.2	3.4	2.2	0.0	2.2	0.0
**Weasel-Hunt**	1.6	0.0	1.5	0.0	1.5	0.0	1.5	0.0
**Wildbird-Butcher**	41.3	52.8*	41.5	51.7	41.7	47.1	41.6	71.4
**Wildbird-Eat**	32.1	34.0	32.1	34.5	32.4	17.6	32.0	71.4**
**Wildbird-Hunt**	11.2	1.9^{**}	11.1	0^{*}	11.0	0.0	10.8	14.3

*=p<0.10 **=p<0.05
